# MALDI Mass Spectrometry Imaging and Semi‐Quantification of Topically Delivered Lactic Acid

**DOI:** 10.1111/srt.13485

**Published:** 2023-10-16

**Authors:** Aaron Cohen, Raphael Legouffe, Junhong Mao, Mathieu Gaudin, David Bonnel

**Affiliations:** ^1^ Personal Care Department of the Colgate‐Palmolive Company Piscataway New Jersey USA; ^2^ Aliri, Parc Eurasanté Loos France

**Keywords:** cationic xylene, chemical peels, derivatization, Franz cell, isotope labeling, stratum corneum, trichloroacetic acid

## Abstract

**Background:**

Lactic acid is a common active ingredient in many topical skincare products; however, measuring its delivery into the skin is challenging due to the presence of a large level of endogenous lactic acid. In this study, matrix‐assisted laser desorption/ionization mass spectrometry imaging (MALDI‐MSI) was used to quantitatively and qualitatively measure the delivery of lactic acid into the skin from a range of topical skincare products.

**Materials and methods:**

Porcine skin samples were treated with various skincare products containing lactic acid. After 24 h, skin samples were sectioned and treated via H&E staining or prepared for MALDI‐MSI using chemical derivatization. Samples were then analyzed by MALDI‐MSI imaging to obtain lactic acid distribution in the entire skin section.

**Results:**

Due to the high level of endogenous lactic acid in the skin, a “triple isotope” of lactic acid (L‐Lactic acid‐^13^C_3_), was needed to provide full resolution from the skin's background signal with MALDI‐MSI. With this approach, the topically delivered lactic acid could be quantitatively and qualitatively analyzed from a variety of skincare products.

**Conclusions:**

The combination of L‐Lactic acid‐^13^C_3_ and MALDI‐MSI was successfully used to quantitatively and qualitatively measure the topical delivery of lactic acid from a variety of skincare products. This approach could be used in future work to better understand the mode of action of lactic acid as an active ingredient in skincare products.

## 1 INTRODUCTION

Lactic acid is a ubiquitous ingredient in the skin care industry, appearing in a variety of products ranging from mass market formulas to higher end premium skincare products.^1^, ^2^ Lactic acid is a component of the natural moisturizing factor (NMF),^3^ and therefore at lower concentrations topically applied lactic acid can enhance the skin's hydration.^4^, ^5^ At higher concentrations, lactic acid breaks down the cohesive units between corneocytes in the stratum corneum^6^ and therefore serves as an effective and widely used peeling agent.^7^, ^8^ Because of its varied uses and wide popularity in the skincare industry, understanding the delivery of lactic acid into the skin is an important area of concern for formulators and other skincare professionals.

Despite the importance of understanding the delivery of lactic acid into the skin, actually measuring the topical delivery of lactic acid poses several challenges. First, lactic acid has minimal UV‐vis absorbance, which prevents analysis using conventional methods of HPLC coupled with a UV‐vis detector.^9^, ^10^ Second, on a broader note, any conventional mode of detection, such as mass spectrometry or various commercially available assay kits, are all severely limited by the presence of a large level of endogenous lactic acid in the skin.^11^, ^12^ Therefore, it is impossible to differentiate between lactic acid that has been delivered into the skin from a topically applied product and lactic acid natively present in the skin without resorting to an isotopic labeling strategy. In two separate examples, the use of [1‐^14^C]‐lactic acid was used to measure the topical delivery of lactic acid.^13^, ^14^ Although the use of a ^14^C‐radiolabeled isotope can indeed differentiate topically applied exogenous lactic from the skin's endogenous lactic, the use of radiolabeled lactic acid can pose safety and storage concerns.^15^ Additionally, the aforementioned experiments failed to provide a detailed spatial resolution of the lactic acid distribution within the skin and at best provided an extremely broad breakdown into regions like the stratum corneum, viable epidermis and dermis. Therefore, it will be of great interest to the skincare industry to develop a method that can detect topically delivered lactic acid in the skin, resolve it from the skin's endogenous lactic acid, and provide a detailed spatial resolution of its delivery.

Based on the aforementioned constraints, MALDI mass spectrometry imaging (MALDI‐MSI) provides many advantages to accurately detect topically applied lactic acid within the skin. MALDI‐MSI was introduced in the 1990s^16^ to detect compounds within tissue sections while preserving its histology. Currently, this technique is routinely used in the pharmaceutical industry as well as the dermatological and cosmetic realms.^17^, ^18^ One main advantage of MALDI‐MSI is the targeting of a molecule's specific mass‐to‐charge ratio (m/z), thereby avoiding the use of fluorescent or radioactive labeling. Recent developments have also enabled the quantification of the compounds targeted by MALDI‐MSI.^19^, ^20^ For these reasons, MALDI‐MSI is a highly promising technique to obtain valuable spatial and quantitative information of a compound of interest in a variety of fields, including but not limited to the skincare industry.

In this manuscript, we describe a method that utilizes MALDI‐MSI to detect topically delivered lactic acid into the skin. The key is the use of a “triple isotope” of lactic acid (L‐Lactic acid‐^13^C_3_), as substitution of all three carbon atoms of the lactic acid molecule with the ^13^C‐isotope in the products of interests was needed to adequately resolve the topically delivered lactic acid from the signal arising from the skin's endogenous lactic acid. Topical delivery of lactic acid was confirmed from both a body lotion containing less than 1% lactic and from three different chemical peels containing 10% or more of lactic acid. For the latter of these products, the detailed spatial resolution provided by MALDI‐MSI helped reveal a potential mode of action of lactic acid delivery, in particular how the presence or absence of trichloroacetic acid (TCA) in the chemical peels impacts lactic acid delivery. Although L‐Lactic acid‐^13^C_3_ has been utilized before in general biological research,^21^, ^22^ this is the first report in which such a stable isotope has been used to measure the topical delivery of lactic acid into the skin. Ultimately, we believe that this application of MALDI‐MSI could gain increased utility for the skincare industry by providing further insights into the wide range of personal care products that incorporate lactic acid as an active ingredient.

## 2 MATERIALS AND METHODS

### 2.1 Materials

2,5‐Dihydroxybenzoic acid (2,5 DHB), trifluoroacetic acid (TFA), trimethylamine (TEA), α,α′‐Dibromo‐o‐xylene, and hematoxylin and eosin (H&E) used for the staining were purchased from Sigma Aldrich (St. Louis, MO, USA). LC‐MS grade methanol (MeOH) and LC‐MS grade water (H_2_O) were purchased from Thermo Fisher Scientific (Germany). Indium tin oxide (ITO) slides were purchased from Delta Technologies (Loveland, CO, USA). Pertex mounting medium was obtained from VWR (Fonteney‐sous‐Bois, France).

L‐Lactic acid‐^13^C and L‐Lactic acid‐^13^C_3_ were both obtained from Sigma‐Aldrich (St. Louis, MO, USA). L‐Lactic acid‐^13^C_3_ (Sigma Aldrich product 746258) had an isotopic purity of >99%. The commercially available skincare products tested included a skincare body lotion with 0.45% lactic acid and three chemical peels with 10% or more of lactic acid designated as chemical peel A, chemical peel B, and chemical peel C. Chemical peel A contained 10% lactic acid and 20% TCA, chemical peel B contained 12% lactic acid and 6% TCA and chemical peel C contained 15% lactic acid and 0% TCA. The TCA levels are mentioned as their hypothesized role in lactic acid delivery will be discussed in a later section. To incorporate L‐Lactic acid‐^13^C_3_, vehicles of the appropriate product were obtained that contained all ingredients except the lactic acid, and then the appropriate amount of the L‐Lactic acid‐^13^C_3_ was post‐added.

### 2.2 Skin sample preparation

Samples were applied to porcine ear skin explants, which were obtained from Animal Technologies (Tyler, TX, USA). Skin samples were shaven and had the subcutaneous fat removed. For the penetration studies, a circular piece of skin with a diameter of roughly 3.5 cm was used. Prior to the study, skin samples were checked for any breakage/defects in the skin; skin samples that had such issues were not used in the study. Penetration studies were conducted on a DHC‐6T transdermal system from Logan Instrument Corp (Somerset, NJ, USA). The receiver fluid, which consisted of pure water, was held at a constant temperature of 37°C. For the lotion, sample was applied to the skin and then rubbed on for 30 s, such that the final mass of product applied was 50 ± 2 mg. The modified body lotion was applied to a single piece of skin, which was subsequently sectioned three times, for three mass spec images. For the peels, 20 mg of product was applied and then gently rubbed on with the rubber end of a syringe for 10 s. Each modified peel was applied to two separate pieces of skin, and each piece of skin was sectioned three times, for a total of six mass spec images per skin sample. For both the body lotion and peels, sample was applied to an area corresponding to approximately the inner 90% of the skin. All penetration studies were run for 24 h.

## 3 MALDI IMAGING

### 3.1 Tissue section preparation

Without any further treatment (i.e., washing or wiping), porcine skin samples (treated and untreated) stored at −80°C were placed inside the cryostat (Leica CM3050S). The temperature of the cryostat was maintained around −20°C. Each tissue was fixed on the cryostat support with a few milliliters of optimum cutting temperature gel (OCT; Qpath freeze gel, VWR, Fontenay‐sous‐Bois, France). For each skin sample, tissue sections at 10 μm of thickness were prepared and collected onto ITO slides for MALDI‐MSI purposes. Additional adjacent sections were collected onto Superfrost slides for H&E staining. Each slide was cryo‐dried inside the cryostat for 15 min. If they were not analyzed immediately, slides were stored at −80°C.

### 3.2 Preparation of the lactic acid calibration curve

For MALDI‐MSI experiments, dilution series (eighteen non‐zero calibration standard levels and one blank) of the test item in LC‐MS grade water were prepared from a stock solution in water. The concentrations of the calibration standards were: 0.1, 0.25, 0.5, 0.75, 1, 2, 2.5, 5, 7.5, 10, 20, 25, 50, 75, 100, 250, and 500 μM, and the resulting calibration curve can be found in the supporting information (Figure S2). One microliter of each solution was spotted near the tissue sections. The slides were then placed in a desiccator for 15 min before MALDI matrix deposition. After analysis of the dilution series at 200 μm of spatial resolution, the sensitivity (Limit of Detection‐LOD, equivalent to Lower Limit of Quantification) of the MALDI‐FTICR method used was calculated as 0.7 μg/g of tissue and the Upper Limit of Quantification (ULOQ) as 146.9 μg/g of tissue. LOD was defined as the last concentration with a signal above the noise and the LLOQ was defined as the first concentration in the linear range.

### 3.3 CAX‐B derivatization

CAX‐B solution was prepared in water/acetonitrile (1:1 v:v) and then sprayed with a HTX TM‐sprayer system (HTX Imaging, Chapel Hill, NC, USA) onto the tissue sections. After that, the slide was incubated in the dark for 30 min at room temperature.

### 3.4 MALDI matrix preparation, deposition and acquisition

2,5‐DHB solution was prepared at 40 mg/mL in methanol/1% trifluoroacetic acid (1:1 v:v).This matrix solution was sprayed with the HTX TM‐sprayer system (HTX Imaging, Chapel Hill, NC, USA) onto the tissue sections. The same method and the same matrix preparation were used for all tissue section analyses. MALDI images were obtained using a 7T MALDI‐FTICR (Solarix, Bruker Daltonics, Bremen, Germany) equipped with a SmartBeam II laser used with a repetition rate of 2000 Hz, in positive ion mode. Mass spectra were acquired with a CASI (Continuous Accumulation of Selected Ion) mode (270–300 Da mass range); the spatial resolution was 100 μm. For each position of the images, the mass spectrum obtained corresponds to the average of 300 consecutive laser shots. FTMS Control 2.2.0 and FlexImaging 5.0 software (Bruker Daltonics, Bremen, Germany) were used to control the mass spectrometer and set the imaging parameters. The test analyte was detected and quantified at the m/z ratio after CAX‐B derivatization (see below table for relevant m/z ratios).

### 3.5 Histological staining

Hematoxylin and Eosin (H&E) staining was performed on the adjacent tissue sections (Superfrost slides). The H&E staining was used to correlate the regions of interest (epidermis and dermis) with the molecular distributions obtained by MSI. High resolution images were generated with the DM6000 microscope (Leica) with 10X magnification.

### 3.6 MALDI imaging data analysis

High‐definition H&E optical images (10x) and MSI data sets were loaded in Multimaging 1.2.6.2 software (Aliri, Lille, France). Then, test item concentrations were calculated for the entirety of each tissue section.

### 3.7 Depth profiling

To analyze the penetration profiles of the test items, a region of interest (ROI) per tissue section was delineated based on its molecular distribution in Multimaging software. This region contained the epidermis and the dermis. For each ROI per tissue, the intensity of the test item per position (i.e., pixel) was extracted from the data set. Then, an alignment of the position through the x axis was performed with Multimaging software defining the 0‐mm value (depth) as the first pixel (spectrum) line (x1y1; x2y1; x3y1…..) obtained in the epidermis.^18^ From the alignment, a new matrix of values (intensity per position) was created, extracted and then used to generate the penetration profile (intensity as a function of depth (y lines) in Excel (Microsoft Office). Finally, the intensities were converted into micrograms per gram of tissue to get a new penetration profile (concentration as function of depth).

## 4 RESULTS

**FIGURE 1:**
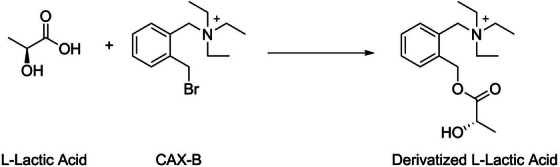
Derivatization reaction of L‐lactic acid with cationic xylene (CAX‐B).

Due to the low molecular weight of lactic acid (90.03 g/mol) and its poor ionization properties with MALDI, chemical derivatization is the preferred route.^23^, ^24^ As shown in Figure 1, CAX‐B (cationic xylene) was used to increase the lactic acid molecular weight and to provide a charge for its detection by MALDI‐MSI. This derivatization technique has been previously described.^25^, ^26^ Importantly, this derivatization process worked equally well for all isotopic forms of lactic acid.

**FIGURE 2:**
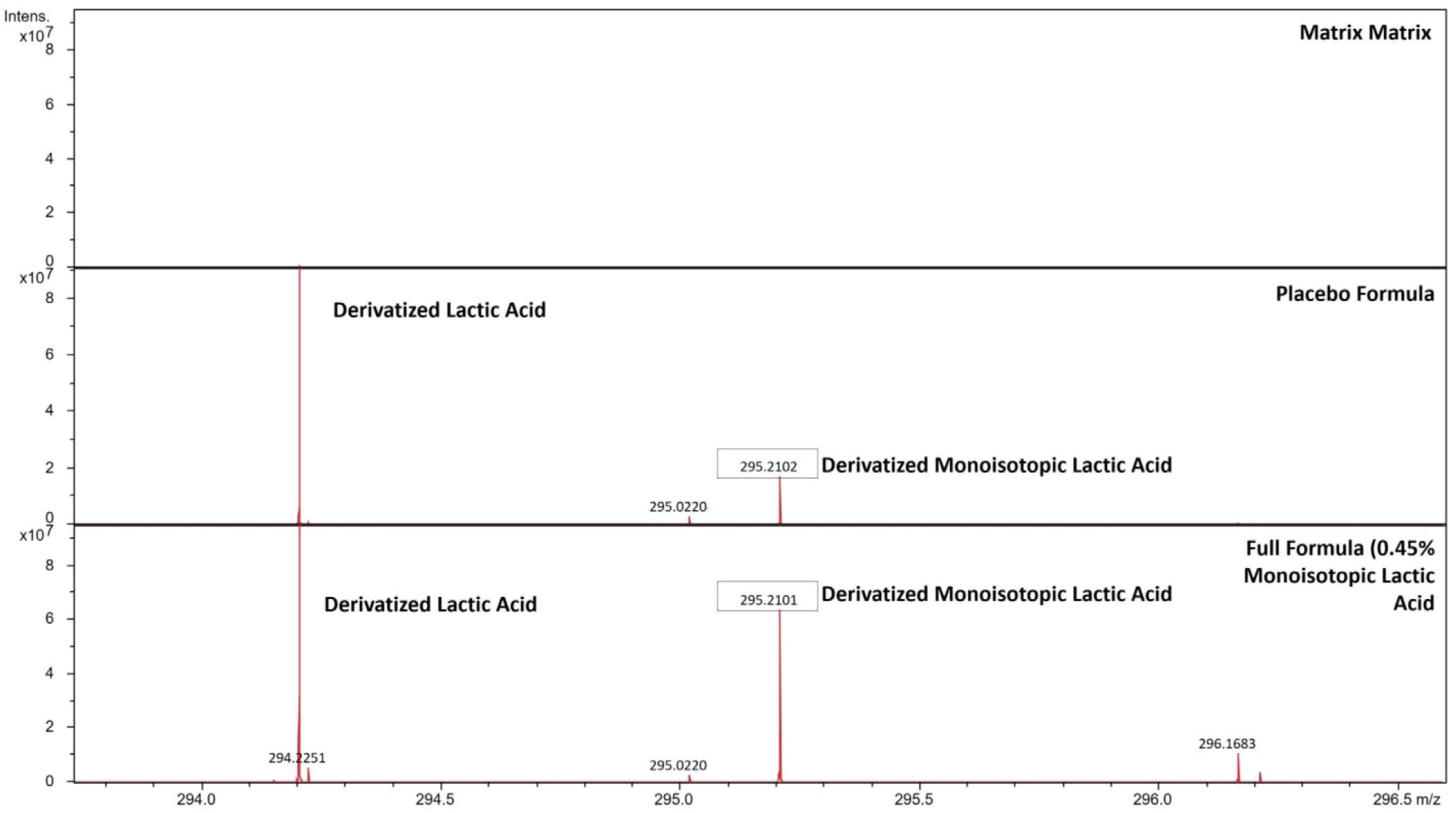
MALDI spectra of matrix only (top row) or porcine skin ear sections treated with either a placebo body lotion containing no lactic acid (middle row) or a body lotion containing 0.45% L‐Lactic acid‐^13^C (bottom row).

Because the skin contains a large level of lactic acid,^11^, ^12^ we utilized a stable ^13^C‐isotope of lactic acid to resolve the topically applied lactic acid from the endogenous lactic acid. L‐Lactic acid‐^13^C, in which a single carbon atom is replaced with the ^13^C‐isotope, was initially tried. Porcine ear skin was treated with either a placebo body lotion (no lactic acid of any form) or a modified body lotion that contained 0.45% L‐Lactic acid‐^13^C. As seen in the MALDI spectra of Figure 2, this approach failed to fully resolve the topically applied lactic acid signal from the signal arising from the skin's endogenous lactic acid. This effect can be understood by analyzing the m/z ratios of the derivatized forms of lactic acid, along with their respective isotopic distributions (Table 1). Because a derivatized form of lactic acid is used, the target molecule has a total of 17 carbon atoms, rather than the three carbon atoms present in lactic acid. Because of this, the M+1 isotopic peak of the skin's endogenous lactic acid will be present at a relatively high abundance of about 19.2% after it undergoes chemical derivatization (see row 1, ‘‘MW isotope 2″ of Table 1).

**TABLE 1:** Mass‐to‐charge (m/z) ratios of the different lactic acid forms, derivatized with cationic xylene (CAX‐B), along with their relevant isotopic distributions.

Compound	Formula	MW isotope 1	MW isotope 2	MW isotope 3	MW isotope 4
Lactic acid	C_17_H_28_NO_3_^+^	294.2064 (100%)	295.2097 (19.188%)	296.2123 (2.357%)	297.2150 (0.257%)
Lactic acid‐^13^C	^13^CC_16_H_28_NO_3_^+^	295.2097 (100%)	296.2130 (18.107%)	297.2156 (2.161%)	298.2182 (0.193%)
Lactic acid‐^13^C_2_	^13^C_2_C_15_H_28_NO_3_^+^	296.2131 (100%)	297.2164 (17.025%)	298.2189 (1.977%)	299.2215 (0.172%)
Lactic acid‐^13^C_3_	^13^C_3_C_14_H_28_NO_3_^+^	297.2164 (100%)	298.2197 (15.944%)	299.2222 (1.804%)	300.2248 (0.152%)

**FIGURE 3:**
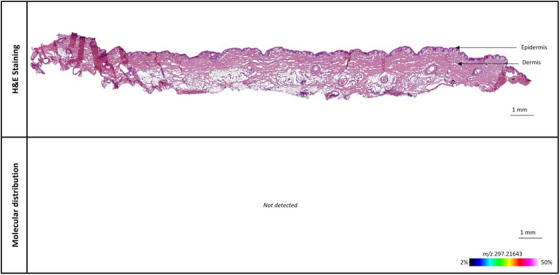
MALDI‐MSI of porcine skin ear skin sample treated with a placebo body lotion (no lactic acid of any isotopic form) and measured for the presence of derivatized L‐Lactic acid‐^13^C_3_ (m/z = 297.2164).

To resolve this issue, we utilized L‐Lactic acid‐^13^C_3_, in which all three carbon atoms of lactic acid are replaced with the ^13^C‐isotope. A mass spectrum showing both derivatized L‐Lactic acid and derivatized L‐Lactic acid‐^13^C_3_ can be found in the supporting information, along with a calibration curve of L‐Lactic acid‐^13^C_3_ on treated tissue sections (Figures S1 and S2, respectively). A porcine ear skin sample treated with a placebo body lotion (no lactic acid of any form) was sectioned and analyzed with MALDI‐MSI for the presence of the derivatized L‐Lactic acid‐^13^C_3_ (m/z = 297.2164, see row 4, ‘‘MW isotope 1″ of Table 1). As seen in Figure 3, no observable signal is seen in this skin sample. This indicates that by using L‐Lactic acid‐^13^C_3_, we can successfully eliminate any contribution from the skin's endogenous lactic acid, thereby providing a suitable pathway to measure the delivery of topically applied lactic acid.

**FIGURE 4:**
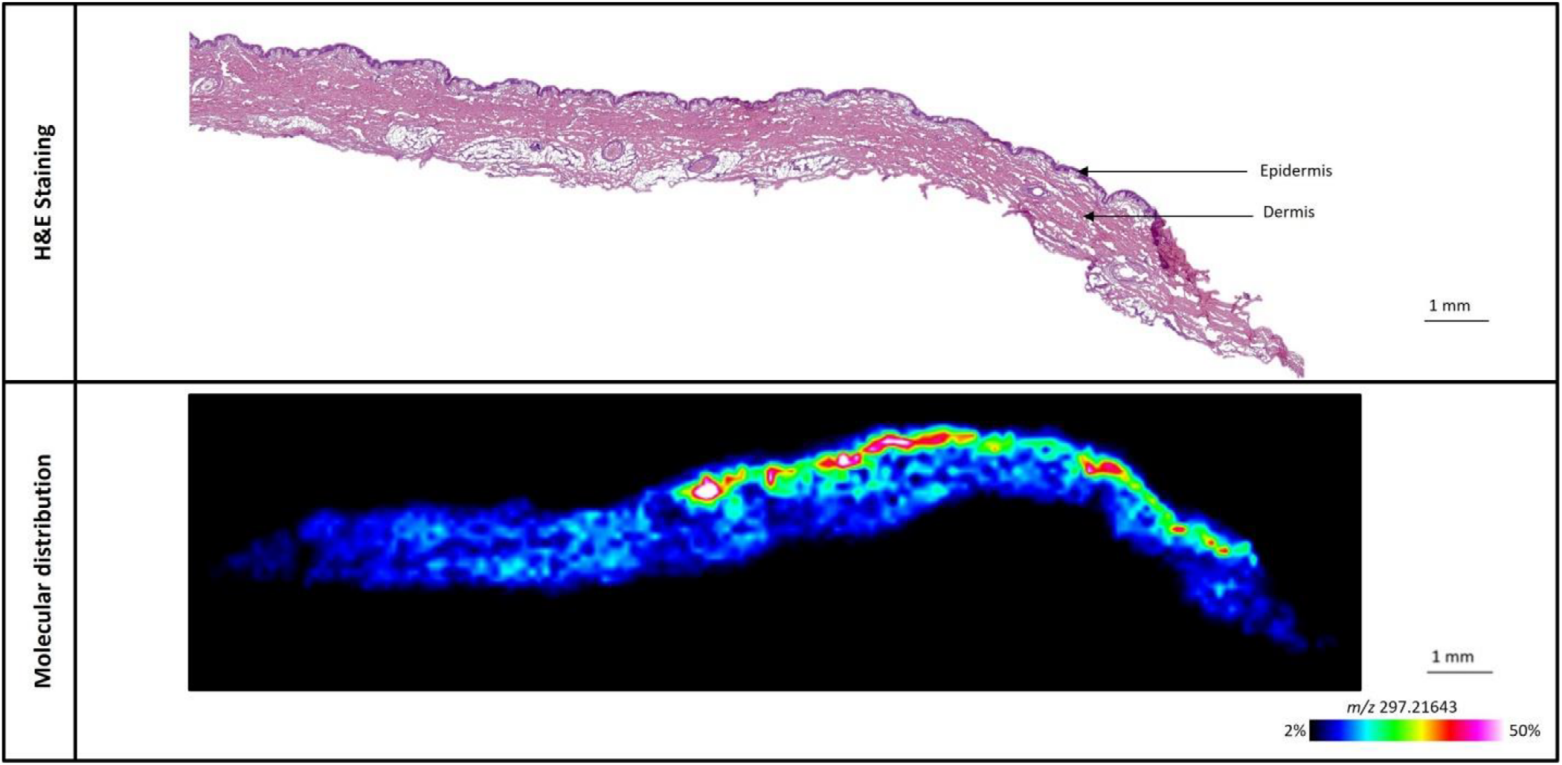
Distribution of L‐Lactic acid‐^13^C_3_ from a modified body lotion containing 0.45% L‐Lactic acid‐^13^C_3_.

This method offers us the ability to evaluate the penetration of topically applied lactic acid from several modified skincare products and to compare the overall delivery of lactic acid between similar formulas. In the first experiment, the topical delivery of L‐Lactic acid‐^13^C_3_ was measured from a simple body lotion that was modified to contain 0.45% of L‐Lactic acid‐^13^C_3_. Porcine ear skin samples were treated with the modified body lotion and placed on a Franz diffusion cell for 24 h, after which they were sectioned and analyzed via MALDI‐MSI. The distribution of L‐Lactic acid‐^13^C_3_ is seen in Figure 4. It can be clearly observed that the majority of the penetrated L‐Lactic acid‐^13^C_3_ is within the topmost regions of the skin, corresponding to the stratum corneum, the viable epidermis, and the upper papillary dermis. This is further confirmed in the distribution profile of the L‐Lactic acid‐^13^C_3_ delivery, which reveals that the majority of the delivered L‐Lactic acid‐^13^C_3_ is within the top most 200 μm of the skin (Figure S3).

Having confirmed that the use of L‐Lactic acid‐^13^C_3_ can distinguish topically applied lactic acid from the skin's endogenous signal, and having measured the delivery of L‐Lactic acid‐^13^C_3_ from a simple body lotion, we next measured the topical delivery of L‐Lactic acid‐^13^C_3_ from three chemical peels. As described above, the three chemical peels all contained between 10%–15% of the L‐Lactic acid‐^13^C_3_ along with varying levels of TCA. The relevant compositional details are seen in Table 2 below.

**TABLE 2:** Composition of modified chemical peels investigated for L‐Lactic acid‐^13^C_3_ delivery.

Sample	L‐Lactic acid‐^13^C_3_ concentration	Trichloroacetic acid (TCA) concentration	Formula pH
Chemical Peel A	10%	20%	0.6
Chemical Peel B	12%	6%	1.1
Chemical Peel C	15%	0%	3.1

**FIGURE 5:**
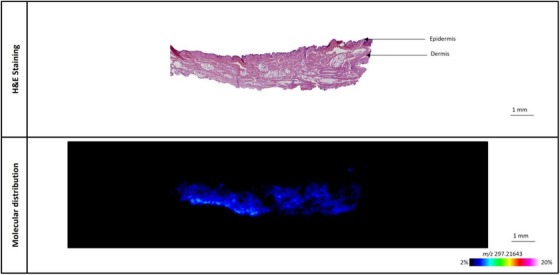
Representative image of L‐Lactic acid‐^13^C_3_ distribution in porcine ear skin sample treated with chemical peel A, containing 10% L‐Lactic acid‐^13^C_3_ and 20% TCA.

**FIGURE 6:**
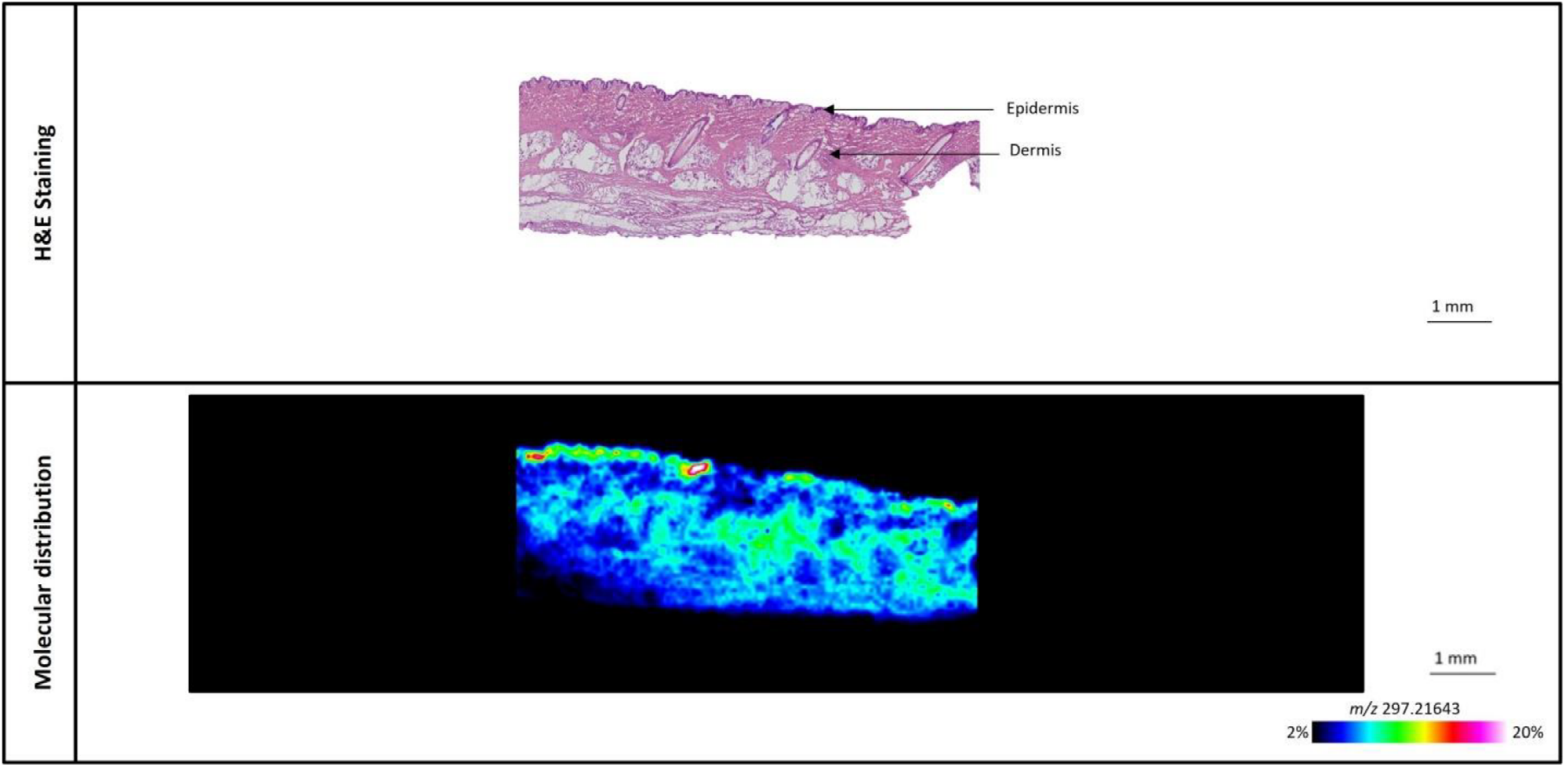
Representative image of L‐Lactic acid‐^13^C_3_ distribution in porcine ear skin sample treated with chemical peel B, containing 12% L‐Lactic acid‐^13^C_3_ and 6% TCA.

**FIGURE 7:**
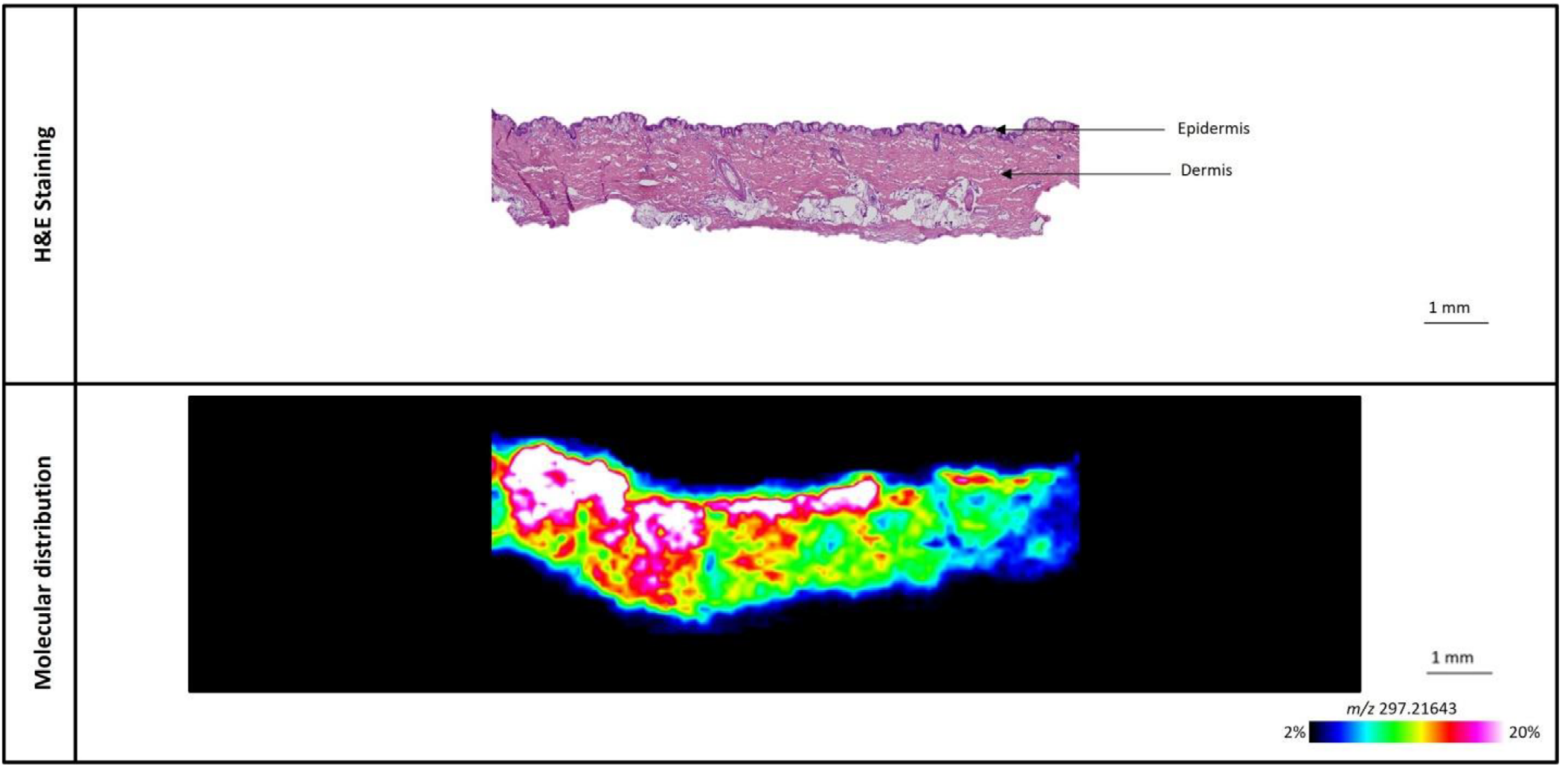
Representative image of L‐Lactic acid‐^13^C_3_ distribution in porcine ear skin sample treated with chemical peel C, containing 15% L‐Lactic acid‐^13^C_3_ and 0% TCA.

The modified chemical peels were applied to porcine ear skin samples and placed on a Franz diffusion cell for 24 h, after which they were sectioned for MALDI‐MSI analysis. Although the level of the L‐Lactic acid‐^13^C_3_ was between 10%–15% for all the chemical peels, the distribution and total delivery of L‐Lactic acid‐^13^C_3_ in the skin differed significantly between all three chemical peels, as illustrated in Figures 5, 6, 7. Despite the highly acidic nature of the chemical peels, H&E imaging of the skin samples revealed no tissue damage to the samples tested (Figures 5, 6, 7).

The distribution of L‐Lactic acid‐^13^C_3_ from chemical peel A is seen in Figure 5. Surprisingly, there is very little L‐Lactic acid‐^13^C_3_ seen within the skin, especially compared to the other two modified chemical peels (see below), despite the fact that this product contained 10% of L‐Lactic acid‐^13^C_3_. The distribution profile of L‐Lactic acid‐^13^C_3_ from one of the skin samples reveals that the small amount of L‐Lactic acid‐^13^C_3_ that was observed within the skin is found primarily in the deeper sections of the skin, about 1–2 mm below the skin surface (Figure S4).

The distribution of L‐Lactic acid‐^13^C_3_ from chemical peels B and C is seen in Figures 6 and 7, respectively. Compared to the distribution of L‐Lactic acid‐^13^C_3_ from chemical peel A (Figure 5), there is an overall greater amount of L‐Lactic acid‐^13^C_3_ seen in both Figures 6 and 7. In Figure 6, there is a more concentrated region of L‐Lactic acid‐^13^C_3_ near the skin surface along with a band of moderate intensity in the middle part of the skin; towards the deeper part of the skin the level of L‐Lactic acid‐^13^C_3_ diminishes. The skin treated with chemical peel C (Figure 7) clearly shows the highest overall level of delivered L‐Lactic acid‐^13^C_3_. Although the signal in Figure 7 is most intense closer to the skin surface, there is a strong overall signal throughout the skin. The distribution and penetration profile of L‐Lactic acid‐^13^C_3_ from skin samples treated with chemical peels B and C can be seen in Figures S5 and S6, respectively.

## 5 DISCUSSION

Lactic acid is present at high levels in the skin and is present in a wide range of skincare products at various levels, where it provides a plethora of skin benefits. Therefore, it is both critical and challenging to accurately measure the topical delivery of lactic acid into the skin. As mentioned in the introduction, there are several reports that use ^14^C‐radiolabeled lactic acid to measure its topical delivery into the skin via scintillation counting.^13^, ^14^ Although this approach can effectively differentiate topically delivered lactic acid from the skin's endogenous lactic acid, the use of radiolabeled ^14^C‐material poses significant hurdles in terms of safety issues.^15^, ^27^, ^28^ Additionally, previous research has shown that the use of a stable ^13^C‐isotope of lactic acid for biological investigations provides comparable results to the use of a radiolabeled ^14^C‐isotope.^21^

In our approach, we combined the use of a ^13^C‐isotope of lactic acid with MALDI‐MSI to both differentiate the topically delivered lactic acid from the skin's endogenous lactic acid and provide a detailed spatial analysis of its overall delivery. The use of L‐Lactic acid‐^13^C, in which a single carbon atom is replaced with the ^13^C‐isotope, proved insufficient to resolve the signals of exogenous and endogenous lactic acid. As illustrated in Table 1, a relatively high level of the derivatized L‐Lactic acid (C_17_H_28_NO_3_^+^) will exist as ^13^CC_16_H_28_NO_3_^+^ owing to the natural abundance of the ^13^C isotope of about 1.1%.^29^ Therefore, simply replacing a single carbon atom of lactic acid with the ^13^C‐isotope will not provide sufficient resolution; the use of L‐Lactic acid‐^13^C_3_, in which all three carbon atoms of lactic acid are replaced with the ^13^C‐isotope, was needed to provide full resolution.

**FIGURE 8:**
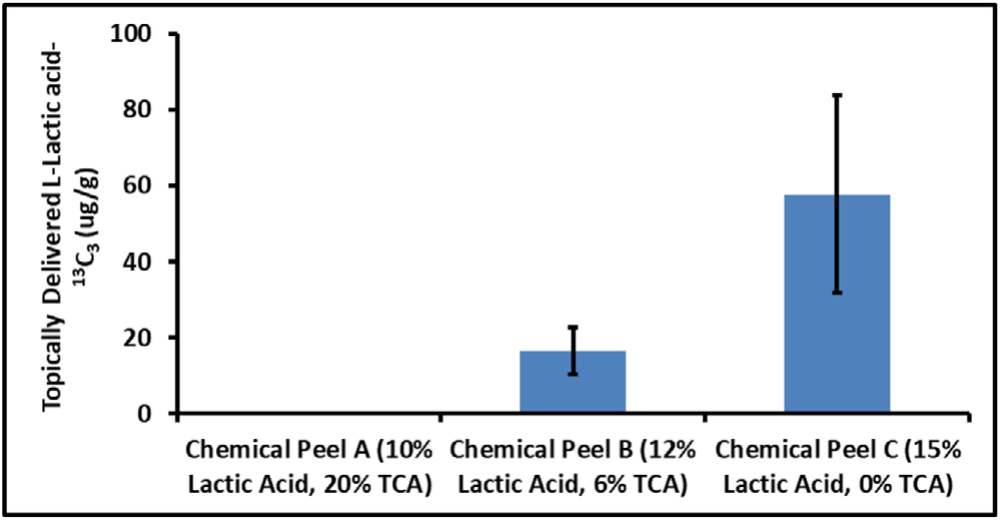
Comparison of L‐Lactic acid‐^13^C_3_ among three different modified chemical peels.

This approach was first tested to measure the topical delivery of 0.45% L‐Lactic acid‐^13^C_3_ from a simple body lotion. For this product, the MALDI‐MSI results and the corresponding distribution profile revealed a gradient of L‐Lactic acid‐^13^C_3_ delivery, with the highest levels of L‐Lactic acid‐^13^C_3_ near the skin surface.^30^ However, a far more complex picture emerged when the topical delivery of L‐Lactic acid‐^13^C_3_ was measured from the three chemical peels. Each chemical peel contained high levels of L‐Lactic acid‐^13^C_3_, (between 10%–15%), and yet the total level of L‐Lactic acid‐^13^C_3_ delivered into the skin after 24 h differed greatly, as seen in Figures 5, 6, 7. This effect is further seen in Figure 8, which displays the total amount of L‐Lactic acid‐^13^C_3_ in the skin in units of μg of L‐Lactic acid‐^13^C_3_/g of tissue. Although Figure 5 clearly indicates a small detectable level of L‐Lactic acid‐^13^C_3_ for skin samples treated with chemical peel A, this level was below our lower limit of quantification (LLOQ) and thus is reported as 0 μg/g in Figure 8.

Even when considering the variation in L‐Lactic acid‐^13^C_3_ concentration among the chemical peels (discussed further below), there appears to be an inverse relationship between the level of TCA in the chemical peels and the level of topically delivered L‐Lactic acid‐^13^C_3_. Chemical peel A, with 20% TCA, has a level of topically delivered L‐Lactic acid‐^13^C_3_ below the LLOQ of our methodology while chemical peel C, with no TCA present, has the highest level of topically delivered L‐Lactic acid‐^13^C_3_. We hypothesize that because the penetration studies were run for 24 h, the TCA effectively acted as a powerful penetration enhancer, heavily or fully impairing the outermost stratum corneum of the skin barrier to allow L‐Lactic acid‐^13^C_3_ to be delivered unimpeded into the skin. TCA operates in part by diminishing corneocyte adhesion,^31^, ^32^ which would weaken or completely eliminate the chief barrier to active delivery into the skin.^33^ 20% TCA in particular will cause significant levels of destruction to the skin barrier, resulting in the well‐known “frosting” effect seen in chemical peels with high levels of TCA.^34^, ^35^ Additionally, TCA is known to cause protein coagulation,^36^, ^37^ which will further accelerate the clearance of actives into and through the skin. For this reason, the L‐Lactic acid‐^13^C_3_ is believed to have had no or very little effective barrier to delivery into the skin for the samples treated with chemical peel A. We speculate that by the time the experiment concluded after 24 h, the majority of L‐Lactic acid‐^13^C_3_ from chemical peel A had cleared the skin and entered the receiver chamber of the Franz diffusion cell. This hypothesis is further supported by the distribution graph of topically delivered L‐Lactic acid‐^13^C_3_ from chemical peel A (Figure S4). In this graph, the small amount of L‐Lactic acid‐^13^C_3_ delivered from chemical peel A is in the deeper part of the skin, about 1–2 mm deep from the skin surface. This suggests that the vast majority of L‐Lactic acid‐^13^C_3_ from chemical peel A had already cleared the skin and only a small amount remained in the deeper part of the skin closest to the receiver chamber of the Franz diffusion cell.

To fully confirm this hypothesis, two key changes would need to be implemented in our experiment. First, as is clearly seen in Table 2, the three modified chemical peels have differing levels of L‐Lactic acid‐^13^C_3_. Although this makes it challenging to directly compare the variations in L‐Lactic acid‐^13^C_3_ delivery, the differences in the overall delivery of L‐Lactic acid‐^13^C_3_ that we observed cannot be wholly attributed to differences in the initial concentrations in the peels. For example, chemical peel C has 25% more L‐Lactic acid‐^13^C_3_ than chemical peel B (15% vs. 12%) and yet the total amount of L‐Lactic acid‐^13^C_3_ delivered into the skin from chemical peel C is more than three times that delivered from chemical peel B (57.7 μg/g vs. 16.5 μg/g respectively).

The second change would be to measure the level of L‐Lactic acid‐^13^C_3_ within the receiver fluid chamber of the Franz cell. In particular, the level and distribution of L‐Lactic acid‐^13^C_3_ from chemical peel A within the skin suggests that there is a large amount of L‐Lactic acid‐^13^C_3_ also present within the receiver fluid; it is also likely that the receiver fluids from skin samples treated with chemical peels B and C contain some level of L‐Lactic acid‐^13^C_3_ simply given the high concentration of L‐Lactic acid‐^13^C_3_ in the starting products and the relatively long experimental time. By measuring the level of L‐Lactic acid‐^13^C_3_ in the receiver fluid along with the corresponding level and distribution of L‐Lactic acid‐^13^C_3_ in the skin sample, we can obtain a complete picture of the overall delivery of L‐Lactic acid‐^13^C_3_ from the chemical peels. Additionally, by measuring the level of L‐Lactic acid‐^13^C_3_ in the receiver fluid, multiple time points can be easily analyzed, providing a more detailed picture of the topical delivery. Such modifications and investigations are the focus of our current work.

## 6 CONCLUSION

A method to qualitatively and semi‐quantitatively measure the delivery and distribution of topically applied lactic acid into the skin was developed using MALDI‐MSI. A key element consists of the use of L‐Lactic acid‐^13^C_3_; due to the derivatization of lactic acid with cationic xylene, all three carbon atoms must be replaced with the ^13^C‐isotope to provide full resolution from the skin's endogenous lactic acid. The method was demonstrated using a modified body lotion and several modified chemical peels. The latter of these products revealed unexpected levels and distribution of L‐Lactic acid‐^13^C_3_ in the skin, which we attribute to the effect of TCA present in several of the peel products. Further exploring this phenomenon is the subject of our current work.

## CONFLICT OF INTEREST STATEMENT

The authors declare no conflicts of interest.

## Supporting information

Supporting Information

## Data Availability

The data that support the findings of this study are available from the corresponding author upon reasonable request.
