# Supplementary material for: MALDI Mass Spectrometry Imaging and Semi‐Quantification of Topically Delivered Lactic Acid
Source: Skin Res Technol. 2023 Oct 16;29(10):e13485. doi: 10.1111/srt.13485 (PMC10579629; doi:10.1111/srt.13485)
Supplement: Supplementary file 1 — Supporting Information [file SRT-29-e13485-s001.docx]

**Supporting Information**

**MALDI Mass Spectrometry Imaging of Topically Delivered Lactic Acid**

**Aaron Cohen^1^, Raphael Legouffe^2^, Junhong Mao^1^, Mathieu Gaudin^2^, and David Bonnel^2^**

**1-Personal Care Department of the Colgate-Palmolive Company, Piscataway, NJ USA**

**2-** **Aliri, Parc Eurasanté, 152 rue du Dr Yersin, 59120 Loos, France**

**
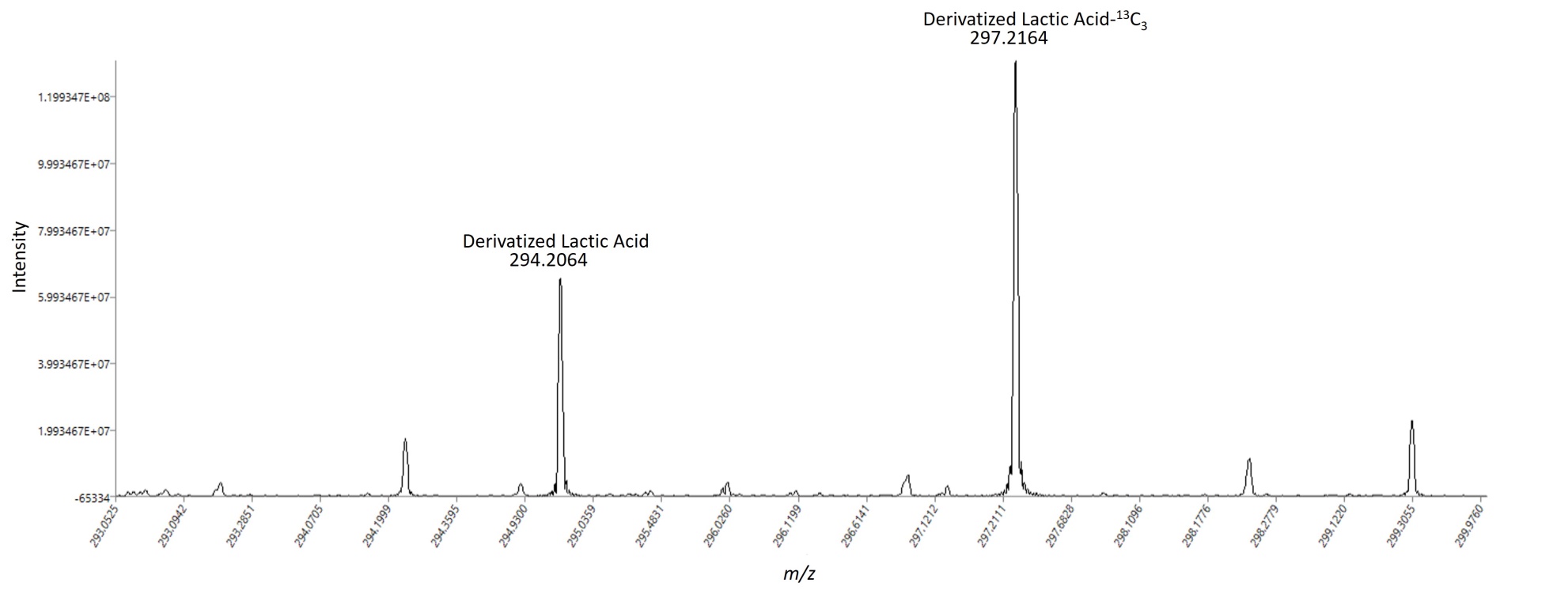
**

**Figure S1:** Mass spectrum showing both derivatized L-Lactic acid and L-Lactic acid-^13^C_3_


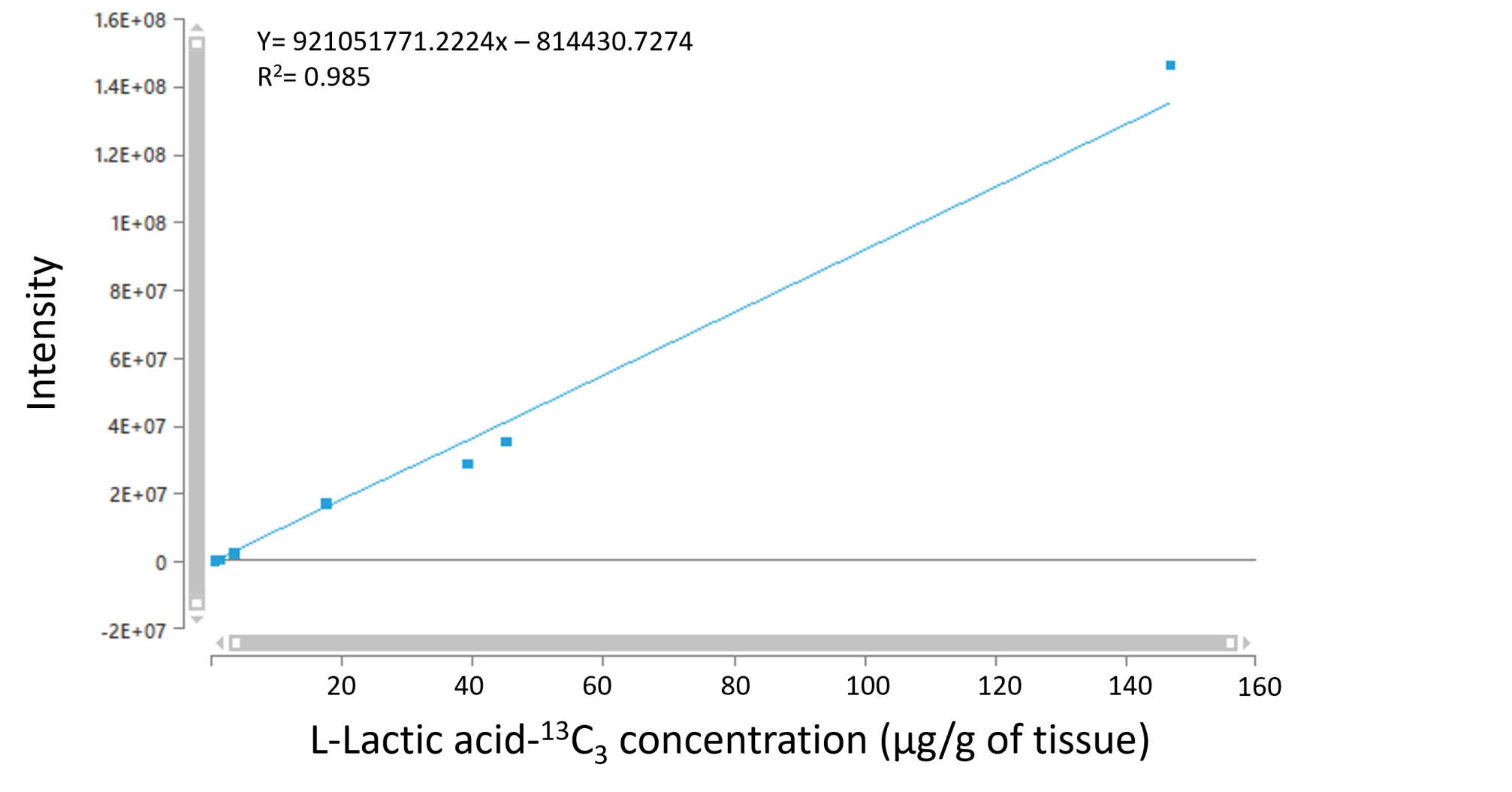


**Figure S2:** Calibration curve of L-Lactic acid-^13^C_3_ on treated tissue sections.


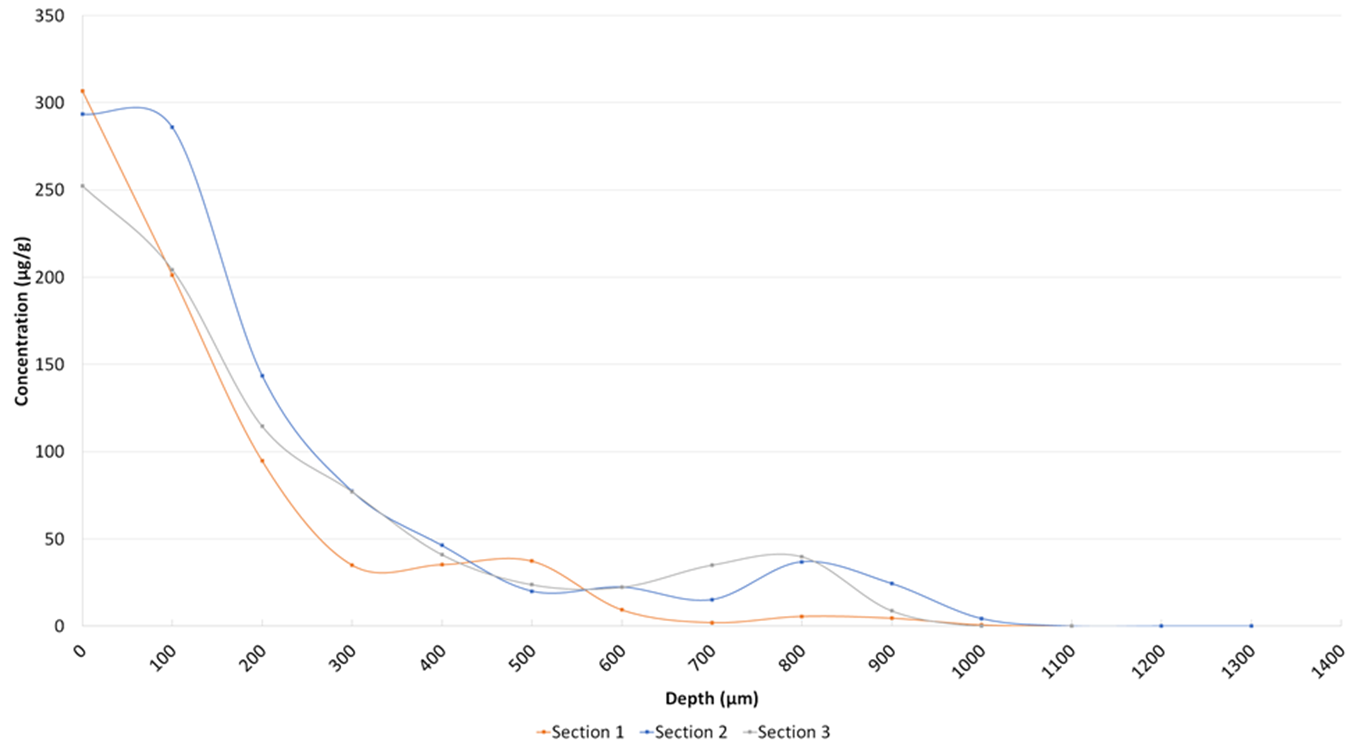


**Figure S3:** Penetration profile of L-Lactic acid-^13^C_3_ from a modified body lotion containing 0.45% L-Lactic acid-13C_3_. Three sections from a single piece of treated porcine skin are shown.


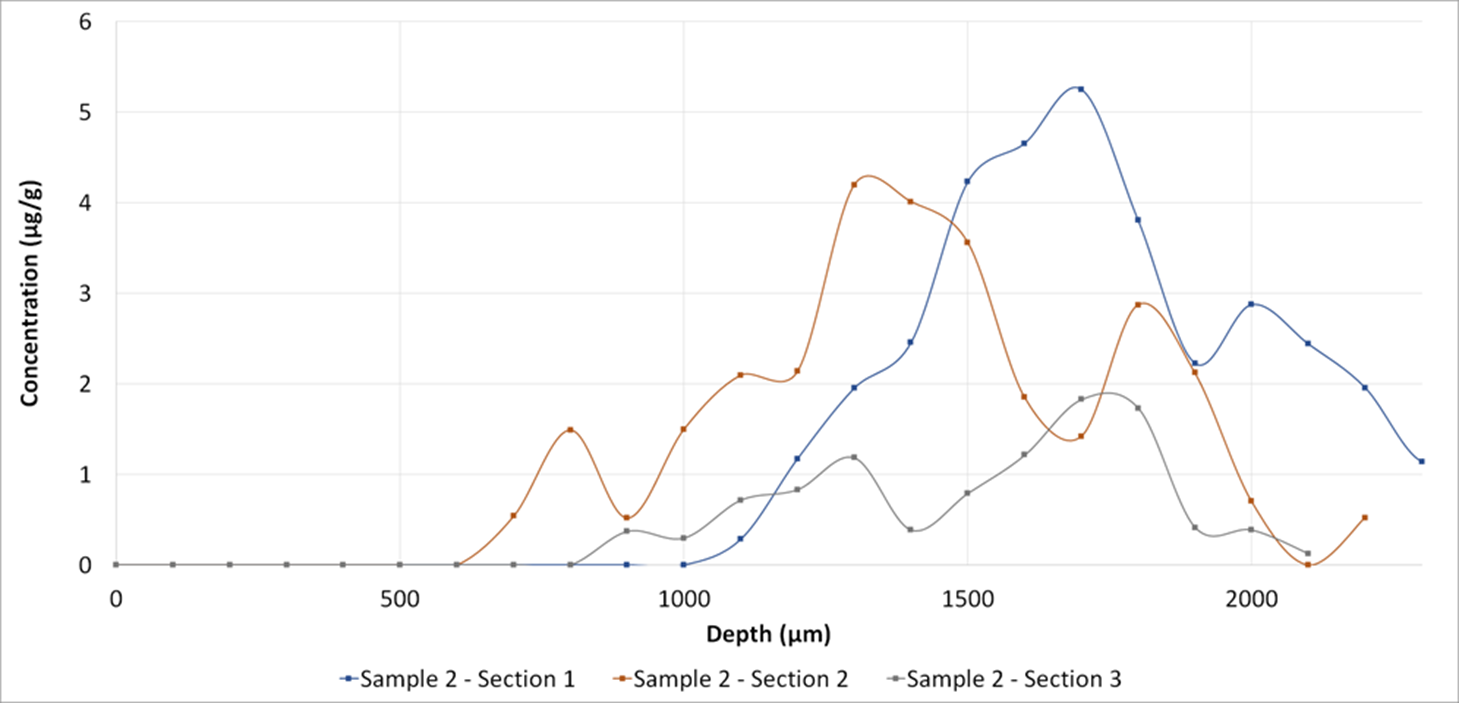


**Figure S4:** Penetration profile of L-Lactic acid-^13^C_3_ from a modified chemical peel containing 10% L-Lactic acid-13C_3_ and 20% trichloroacetic acid. Three sections from a single piece of treated porcine skin are shown.


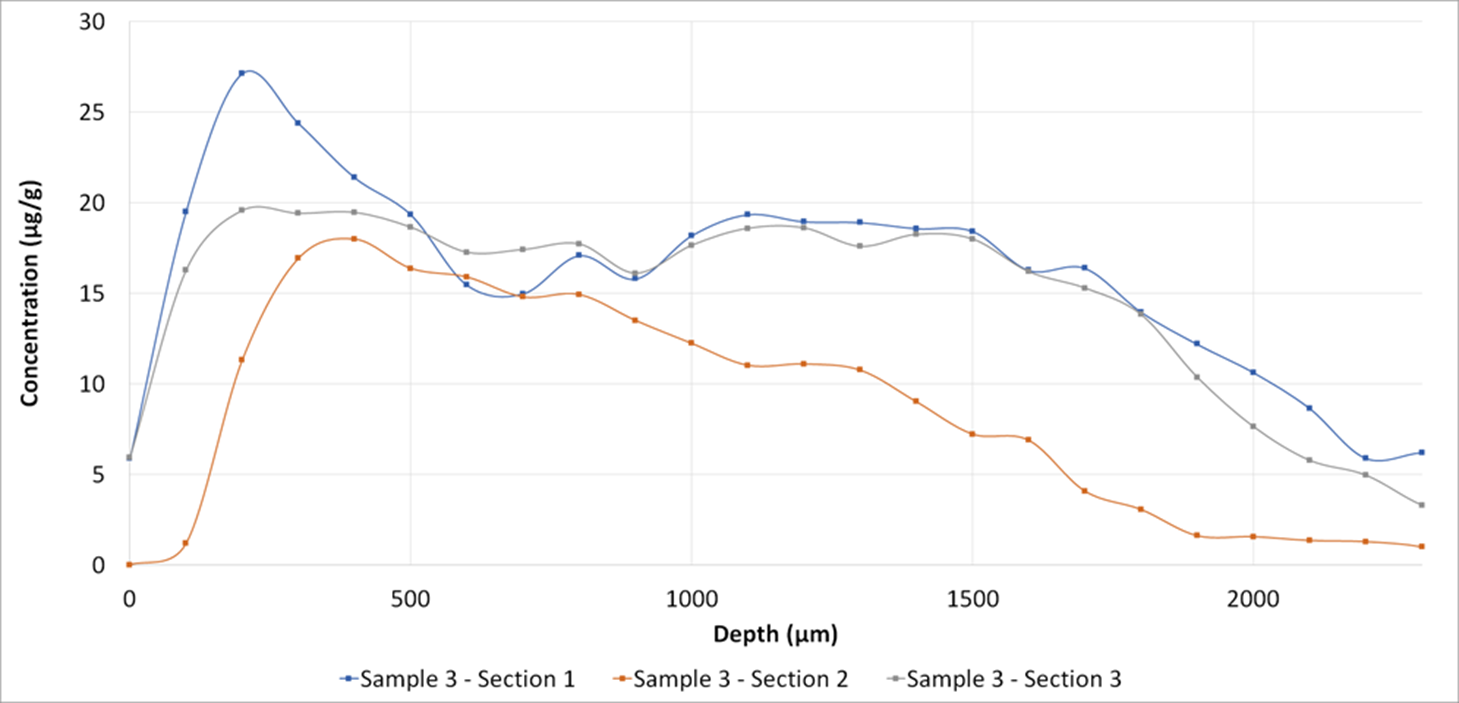


**Figure S5:** Penetration profile of L-Lactic acid-^13^C_3_ from a modified chemical peel containing 12% L-Lactic acid-13C_3_ and 6% trichloroacetic acid. Three sections from a single piece of treated porcine skin are shown.


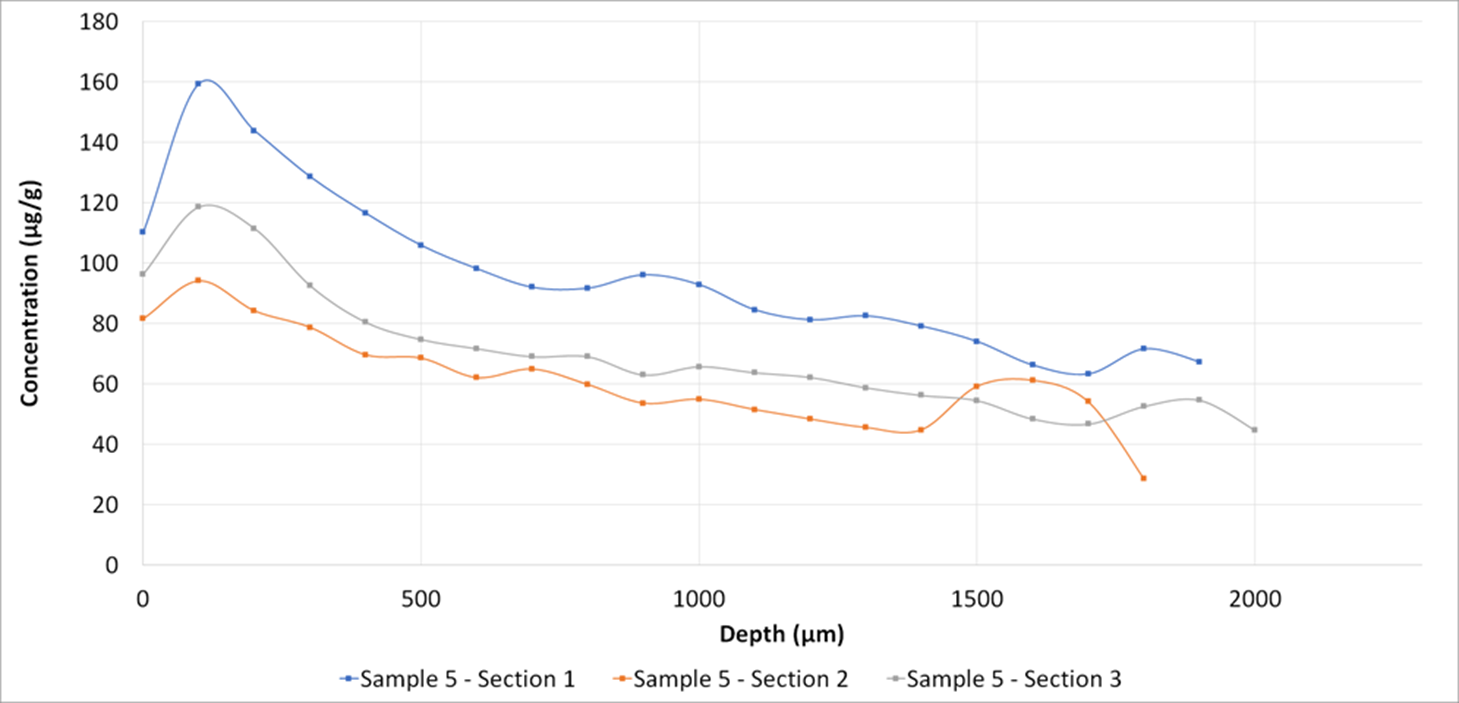


**Figure S6:** Penetration profile of L-Lactic acid-^13^C_3_ from a modified chemical peel containing 15% L-Lactic acid-13C_3_ and 0% trichloroacetic acid. Three sections from a single piece of treated porcine skin are shown.
